# Analysis of genetic diversity and population structure of *Babesia gibsoni*

**DOI:** 10.3389/fvets.2023.1147958

**Published:** 2023-03-23

**Authors:** Fangyuan Yin, Chuanjiang Guo, Zhuojia Tian, Dong Li, Daoe Mu, Haoting Liu, Guiquan Guan, Hong Yin, Facai Li

**Affiliations:** ^1^College of Veterinary Medicine, Southwest University, Chongqing, China; ^2^State Key Laboratory of Veterinary Etiological Biology, Lanzhou Veterinary Research Institute, Chinese Academy of Agricultural Sciences, Lanzhou, Gansu, China

**Keywords:** *Babesia gibsoni*, genetic diversity, population structure, gene flow, 18S rRNA gene

## Abstract

*Babesia gibsoni* is a tick-borne apicomplexan protozoan causing canine babesiosis. This parasite has diploid sexual reproduction in ticks, during which genetic exchanges can occur leading to increased genetic diversity, which is an important factor in adapting to environmental changes. Exploring the genetic variation of *B. gibsoni* population can provide a foundation for understanding the patterns of disease transmission and developing babesiosis control strategies. Partial 18S rRNA fragment sequences were obtained from 11 *B. gibsoni* isolates collected from different regions in China and 117 publicly available sequences were from 12 geographical areas including China. The genetic variation, demographic expansion and population structure were examined. A total of 34 haplotypes were identified among *B. gibsoni* populations. Analysis of molecular variance, pairwise *Fst* and structure analysis showed that high genetic variation within populations, low genetic differentiation and obvious mixture haplotype were apparent in a single continent, but higher genetic differentiation was detected across different continents. Neutrality tests implied that *B. gibsoni* populations had experienced population extension. These findings will contribute to understand the genetics and evolution of *B. gibsoni* and will be useful for formulating effective management strategies to prevent and control this parasite.

## Introduction

Vector-borne pathogens such as malaria, leishmaniasis and trypanosomosis affecting humans and babesiosis, theileriosis and hepatozoonosis in animals are globally important threat factors for health (1). *Babesia gibsoni*, one of tick-borne intracellular hemoprotozoan parasites, is considered to be an important pathogen responsible for wild and domestic canine babesiosis. This parasite is transmitted mainly by *Haemaphysalis longicornis* and *Rhipicephalus sanguineus*, but other sources of transmission to dogs have been demonstrated including dog fighting, blood transfusion and transplacental transmission (2–4). The clinical manifestations of *B. gibsoni* infection include acute hemolysis with lethargy, jaundice, anemia, thrombocytopenia, hemoglobinuria and splenomegaly (5). In chronic infections, the dogs are commonly asymptomatic and become pathogen carriers (6). *B. gibsoni* has a complex life cycle, undergoing gamogony in the midgut lumen of tick, sporogony in the salivary glands of tick and merogony in the blood cells of the vertebrate host (7, 8). This parasite was detected in India in 1910, then successively found in certain parts of Asia, including Bangladesh, China, Japan, Korea, Malaysia, Myanmar, and Thailand (9–18). With the increase in the number and international movement of dogs, *B. gibsoni* has gradually spread around the world to Africa, America, Australia and Europe (19–25).

Understanding the genetic differentiation and population structure of protozoan parasites will be helpful to evaluate their evolution, spatiotemporal dynamics and genetic exchange, as well as to formulate the effective strategies to prevent the transmission of pathogens (26). Molecular markers such as 18S rRNA, mitochondrial DNA, microsatellite loci and single nucleotide polymorphisms are often employed to explore the genetic variation and provide the information on reproductive modes and molecular sequences obtained from samples to explain the evolutionary history of populations (27). Several genetic diversity studies of tick-transmitted hemoprotozoan parasites have currently been described, including *Babesia bovis, Babesia ovis, Hepatozoon canis, Theileria annulata*, and *Theileria parva* (27–31). These studies demonstrated that high genetic diversity was detected in *B. bovis, B. ovis, T. annulata*, and *T. parva* populations (28–31). A high gene flow was found between *H*. *canis* populations originating from distant continents (27). The phylogenetic and population differentiation of *Babesia* spp., which can parasitize cattle, sheep and horses, were explained by previous studies (28, 29, 32). Molecular detection of *B. gibsoni* has been broadly described around the world (20, 22, 33–36), but the genetic diversity of *B. gibsoni* in dogs was scarce.

The present study aimed to explain the genetic differentiation, population dynamics and population structure of *B. gibsoni* populations from Asia, America and Europe using ribosomal RNA 18S (18S rRNA) gene. These findings will help to understand the distribution and dynamics of genetic difference among *B. gibsoni* populations, which is necessary for designing effective control strategies to manage and prevent this parasite.

## Materials and methods

### Sample collection and DNA extraction

A total of 197 blood samples were collected from dogs in pet clinics from five cities, including Chongqing, Linyi, Quanzhou, Wuhan and Xiangyang, as seen in Supplementary Table 1. Most of the samples were asymptomatic for babesiosis, but two dogs showed fever, lethargy, diarrhea and anemia. Approximately 300 μl blood samples were collected in EDTA vacutainers and transported in iceboxes to the College of Veterinary Medicine, Southwest University. Genomic DNA samples were extracted from 250 μl blood by the Blood DNA Mini Kit (Omega, USA), stored at −20°C.

### DNA amplification and sequencing

The presence of DNA from *Babesia* spp. was detected by a nested polymerase chain reaction (PCR). A region of the 18S rRNA gene fragment 1,379 bp in length was amplified using Piro1-S: 5′-CTTGACGGTAGGGTATTGGC-3′, Piro3-AS: 5′-CCTTCCTTTAAGTGATAAGGTTCAC-3′ and then a small fragment 405 bp in length was amplified using Piro-A: 5′-ATTACCCAATMCBGACACVGKG-3′ and Piro-B: 5′-TTAAATACGAATGCCCCCAAC-3′ in the second round of PCR (37, 38). Briefly, PCR was performed in 25 μl containing 2.5 μl of 10 × PCR buffer, 2.0 μl of 2.5 mM dNTP, 0.3 μl of 5 U/μl *Taq* DNA polymerase (Takara, China), 0.1 μM of primer pair and 2 μl of DNA template. The PCR reaction conditions were initial denaturation at 95°C for 3 min, followed by 35 cycles of denaturation at 95°C for 30 s, annealing at 55°C for 30 s and extension at 72°C for 90 s, with a final extension of 72°C for 5 min. Positive products were cloned into pMD19-T vector (TaKaRa, China) and transformed into *Escherichia coli* Trans5α competent cells (TransGen, China). The positive clones were then sequenced by M13-F/M13-R primers (PRISM3730XL, ABI). DNA isolated from blood of a dog infected with *B. gibsoni* and distilled water were used as positive and negative control, respectively.

### Sequences analysis and selection

The 18S rRNA sequences were analyzed using the NCBI BLASTn program (https://blast.ncbi.nlm.nih.gov) and deposited in GenBank under the accession numbers OM392053-OM392060 and ON810382-ON810384.

A total of 117 18S rRNA sequences of *B. gibsoni* were downloaded from GenBank database. Sequences without annotation such as host or location and truncated sequences were excluded from the analysis. All of the sequences were aligned with a consensus length of 409 bp and these sequences belonged to 12 areas from three continents. The details of all the sequences used in this study are shown in Supplementary Table 2.

### Data analysis

Multiple sequences were aligned using the program Clustal W within MEGA11 (39). Genetic parameters including the number of haplotypes, haplotype diversity and nucleotide diversity were performed using DnaSP 5.1 (40). Pairwise *Fst* and *Nst* values were conducted by the DnaSP 5.1 to evaluate the genetic differentiation between pairs of populations. *Fst* values indicate the level of genetic differentiation (low is between zero and 0.05, moderate is between 0.05 and 0.15 and high is between 0.15 and 0.25). Tajima's *D* (41), Fu's *Fs* (42) and pairwise mismatch distribution analysis were performed to test neutrality using the same program (DnaSP 5.1). Tajima's *D* and Fu's *Fs* positive values indicate no deviation from neutrality, which is representative of stationary populations, whereas negative values indicate populations with recent expansion or purifying selection. Populations with only one sample were excluded in Asian countries for the above analyses. To evaluate relationships among haplotypes, a TCS network was drawn using PopArt 1.7 program (43, 44).

The hierarchical analysis of molecular variance (AMOVA) was analyzed to assess the genetic variation within and among *B. gibsoni* populations using Arlequin 3.5 (45). For this analysis, the populations from Asia or the three continents were treated as a single group and the populations from the three continents were divided into two groups, one from Asia and the other from America and Europe.

Population genetic structure was deduced by Structure v2.3.4 (46) using the admixture ancestry model. Each value of *K* (*K* = 1–10) was run with 10 iterations based on the Bayesian Markov Chain Monte Carlo approach. The optimal number of clusters (*K*) were inferred by Δ*K* using the online program Structure Harvester (47, 48). The clusters were shown using CLUMPP v1.1.2 (49) and DISTRUCT 1.1 programs (50). Discriminant analysis of principal components (DAPC) was applied to further identify the genetic clusters of *B. gibsoni* populations based on pre-defined groups using the *adegenet* package (51) implemented in the R software (52). For DAPC, the function “*find. clusers*” was employed to confirm the optimal number of clusters (53). The Mantel test was used to assess the relation between genetic distance and geographical distance by using zt software (54). Populations with only one haplotype were excluded in Asian countries for the above analyses.

## Results

### Molecular detection and identification of *Babesia* spp.

The results indicated that the positive rate of *Babesia* spp. was 5.6%, as seen in Supplementary Table 1. The positive rate of *Babesia* spp. infection was 3.6, 4.4, 14.7, and 10.0% in Chongqing, Quanzhou, Wuhan, and Xiangyang, respectively, as seen in Supplementary Table 1. The obtained sequences were 99.3–99.8% identical with *B. gibsoni* isolates from China (MN928826) by BLAST. The two sick dogs were identified as *B. gibsoni* positive. In total, 11 partial 18S rRNA sequences of *B. gibsoni* were used for the following analysis.

### Genetic diversity analysis and haplotype network

**Figure 1:**
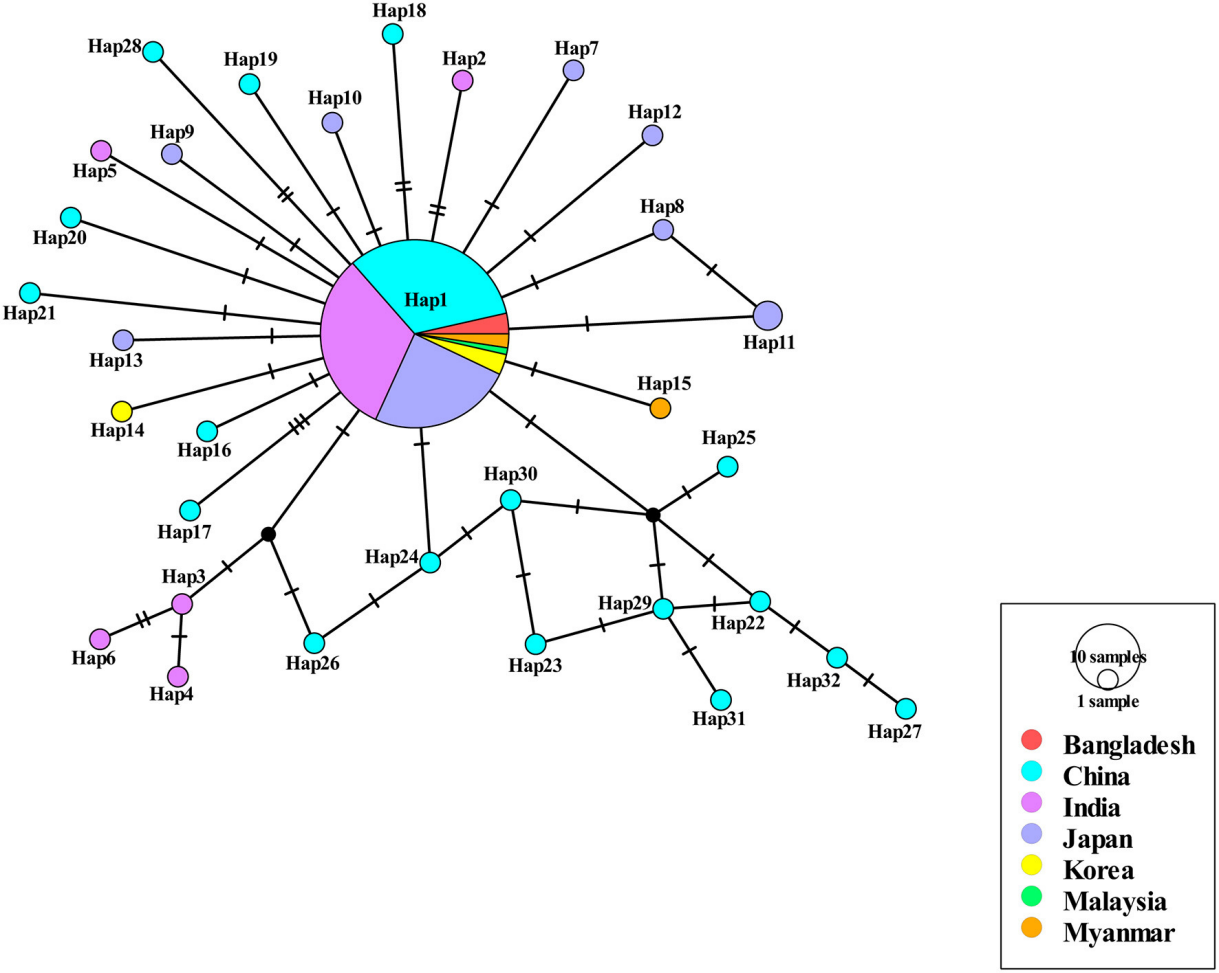
Haplotype network of *Babesia gibsoni* populations in Asia. Haplotype Hap1 containing 85 *B. gibsoni* isolates from seven countries including Bangladesh, China, India, Japan, Korea, Malaysia, and Myanmar. The size of the circle represents the frequency of each haplotype. The different colored dots represent haplotypes from the different populations.

Among the 409 aligned nucleotide sites, 32 variable sites were detected. Of 34 distinct haplotypes identified in 128 individuals, 31 were private haplotypes, as seen in Table 1. Haplotype Hap1 was most common and widespread, distributed in 93 *B. gibsoni* isolates from 12 areas, as seen in Table 1. Among different *B. gibsoni* populations from Asian countries, the haplotype diversity and nucleotide diversity ranged from 0.000 to 0.667 and from 0.0000 to 0.0035, respectively, as shown in Table 2. The highest haplotype diversity was found in Myanmar and the highest nucleotide diversity was found in China. The lowest haplotype and nucleotide diversity were found in Bangladesh, with only one haplotype (Hap1). Globally, the mean haplotype and nucleotide diversity were 0.473 and 0.0022, respectively, as shown in Table 2. The haplotype network showed that there was a low level of sequence differentiation and high frequency of unique mutations among populations, as seen in Figure 1 and Supplementary Figure 1.

**Table 1:** Geographic origin of *Babesia gibsoni* isolates and the number of haplotypes.

**Locations**	**Continents**	**No. of sequences**	**No. of haplotypes**
Bangladesh	Asia	3	1, Hap1 (3)
China	Asia	45	18, Hap1 (28), Hap16 (1), Hap17 (1), Hap18 (1), Hap19 (1), Hap20 (1), Hap21 (1), Hap22 (1), Hap23 (1), Hap24 (1), Hap25 (1), Hap26 (1), Hap27 (1), Hap28 (1), Hap29 (1), Hap30 (1), Hap31 (1), Hap32 (1)
India	Asia	32	6, Hap1 (27), Hap2 (1), Hap3 (1), Hap4 (1), Hap5 (1), Hap6 (1)
Japan	Asia	29	8, Hap1 (21), Hap7 (1), Hap8 (1), Hap9 (1), Hap10 (1), Hap11 (2), Hap12 (1), Hap13 (1)
Korea	Asia	4	2, Hap1 (3), Hap14 (1)
Malaysia	Asia	1	1, Hap1 (1)
Myanmar	Asia	3	2, Hap1 (2), Hap15 (1)
St Kitts	America	1	1, Hap1 (1)
USA	America	5	2, Hap1 (4), Hap34 (1)
Italy	Europe	2	1, Hap1 (2)
Serbia	Europe	2	1, Hap33 (2)
Spain	Europe	1	1, Hap1 (1)
Total	-	128	34

The number of each haplotype is in parentheses.

**Table 2:** Genetic parameters and neutrality tests of *Babesia gibsoni* populations.

**Locations**	***N***	**Hd**	**π**	***D***	***Fs***
**Asian countries**					
Bangladesh	3	0	0	0	0
China	45	0.618	0.0035	−2.264^**^	−15.935^***^
India	32	0.292	0.0018	−1.900^*^	−2.564
Japan	29	0.480	0.0014	−2.073^*^	−7.058^**^
Korea	4	0.500	0.0013	−0.612	0.172
Myanmar	3	0.667	0.0017	n.d.	n.d.
**Continents**					
America	6	0.333	0.0008	−0.933	−0.003
Asia	117	0.474	0.0023	−2.589^***^	−33.791^***^
Europe	5	0.600	0.0015	1.225	0.626
Global	128	0.473	0.0022	−2.610^***^	−54.266^***^

*N*, number of individuals; *Hd*, haplotype diversity; π, nucleotide diversity; *D, Tajima's D*; *Fs, Fu's Fs*; n.d., not detected.

* *p* < 0.05;

** *p* < 0.01,

*** *p* < 0.001.

### Neutrality and demographic analysis

Tajima's *D* and Fu's *Fs* values were significantly negative for China and Japan, indicating a recent population expansion. When Asian populations were considered as a single group, the Tajima's *D* and Fu's *Fs* values were also significantly negative, revealing that the population had experienced recent expansion, as seen in Table 2. The demographic expansion was supported by the pairwise mismatch distribution analysis, as seen in Supplementary Figure 2. The populations from the three continents had also undergone recent expansion as determined by Tajima's *D* and Fu's *Fs* analyses with statistically significant negative values and a star-like haplotype network, as seen in Table 2 and Supplementary Figure 1.

### Population genetic differentiation

The Asian population differentiation is described in Table 3. The results indicated that low genetic differentiation was observed between all pairs of populations, with *Fst* values ranging from 0.000 to 0.0585 and *Nst* values ranging from 0.000 to 0.0578, as shown in Table 3. Compared with other populations, the Chinese population showed the highest genetic differentiation with *Fst* values ranging from 0.0403 to 0.0585 and *Nst* values from 0.0405 to 0.0578, as seen in Table 3. The *Fst* and *Nst* values were also conducted between samples from Asia and those from America and Europe. The comparison results revealed that the highest differentiation was observed between America and Europe with an *Fst* value of 0.1765 and an *Nst* value of 0.1768, whereas the lowest differentiation was obtained between America and Asia with an *Fst* value of 0.0131 and an *Nst* value of 0.0130, as seen in Table 4.

**Table 3:** Pairwise *Fst* and *Nst* values between *Babesia gibsoni* populations in Asia.

**Locations**	**Bangladesh**	**China**	**India**	**Japan**	**Korea**	**Myanmar**
Bangladesh	-	0.0578	0.0320	0.0175	0.0000	0.0000
China	0.0585	-	0.0426	0.0474	0.0437	0.0405
India	0.0323	0.0425	-	0.0264	0.0197	0.0176
Japan	0.0179	0.0474	0.0262	-	0.0096	0.0084
Korea	0.0000	0.0437	0.0194	0.0094	-	0.0005
Myanmar	0.0000	0.0403	0.0171	0.0081	0.00000	-

*Nst* values are above the diagonal and *Fst* values are below the diagonal.

**Table 4:** Pairwise *Fst* and *Nst* values between *Babesia gibsoni* populations in three continents.

**Locations**	**America**	**Asia**	**Europe**
America	-	0.0130	0.1768
Asia	0.0131	-	0.1212
Europe	0.1765	0.1210	-

*Nst* values are above the diagonal and *Fst* values are below the diagonal.

The AMOVA indicated that most genetic variation occurred within populations, when either Asian countries or the three continents were treated as a single group, as shown in Table 5. The hierarchical AMOVA showed that there was much less variation among groups and among populations within groups than within populations, which was supported by the low *F*_*CT*_ and *F*_*SC*_ values with no statistical significance, as shown in Table 5. These findings illustrated low genetic differentiation and high gene flow among populations.

**Table 5:** Analysis of molecular variance (AMOVA) of *Babesia gibsoni* populations.

**Variance component**	**Variance**	**% of total**	***F*-statistic**	***P*-value**
**Asian countries**				
One group^a^				
Among populations	0.0021	0.45		
Within populations	0.4603	99.55	*F_*ST*_* = 0.0045	0.35
**Three continents**				
One group^b^				
Among populations	0.0105	2.10		
Within populations	0.4910	97.90	*F_*ST*_* = 0.0210	0.30
Two groups^c^				
Among groups	−0.0074	−1.48	*F_*CT*_* = −0.0148	0.67
Among populations within groups	0.0178	3.54	*F_*SC*_* = 0.0349	0.19
Within populations	0.4910	97.93	*F_*ST*_* = 0.0207	0.33

a Populations from Asian countries are ungrouped.

b Populations from the three continents are ungrouped.

c Populations from the three continents were divided into two groups, including (Asia) and (Europe and America).

### Population structure analysis

**Figure 2:**
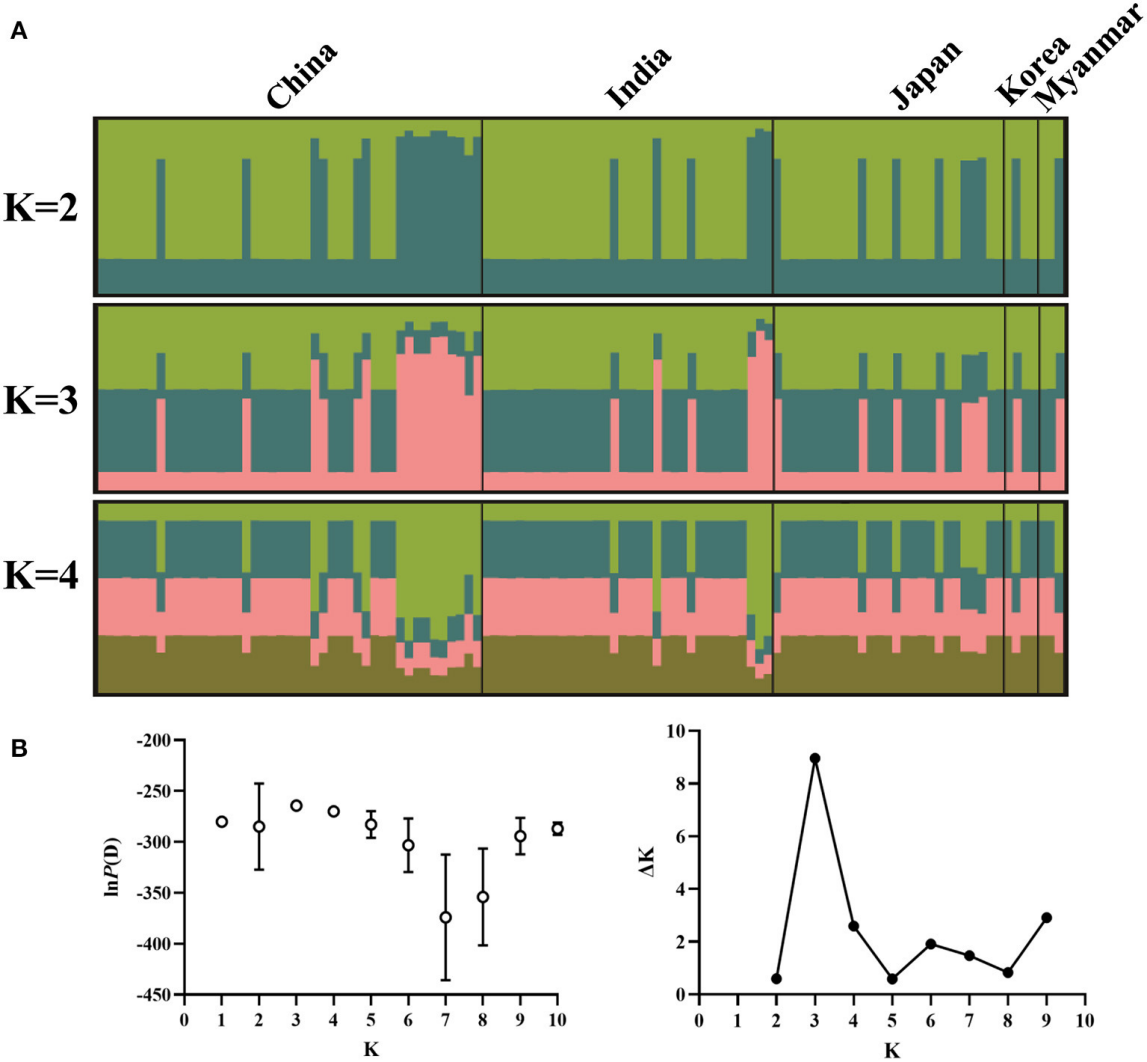
Genetic structure of *Babesia gibsoni* populations in Asia based on Bayesian cluster analysis. **(A)** The clusters are *K* = 2, *K* = 3, and *K* = 4. Different colors represent proportions of five populations in each inferred clusters and the sample name are given above. **(B)** Changes of the *lnP(D)* (mean ± SD) for *K*-values from 1 to 10 and the relationship between delta *K* and *K* values. When *K* = 3, delta *K* has the maximum value, indicating the true number of clusters is three.

**Figure 3:**
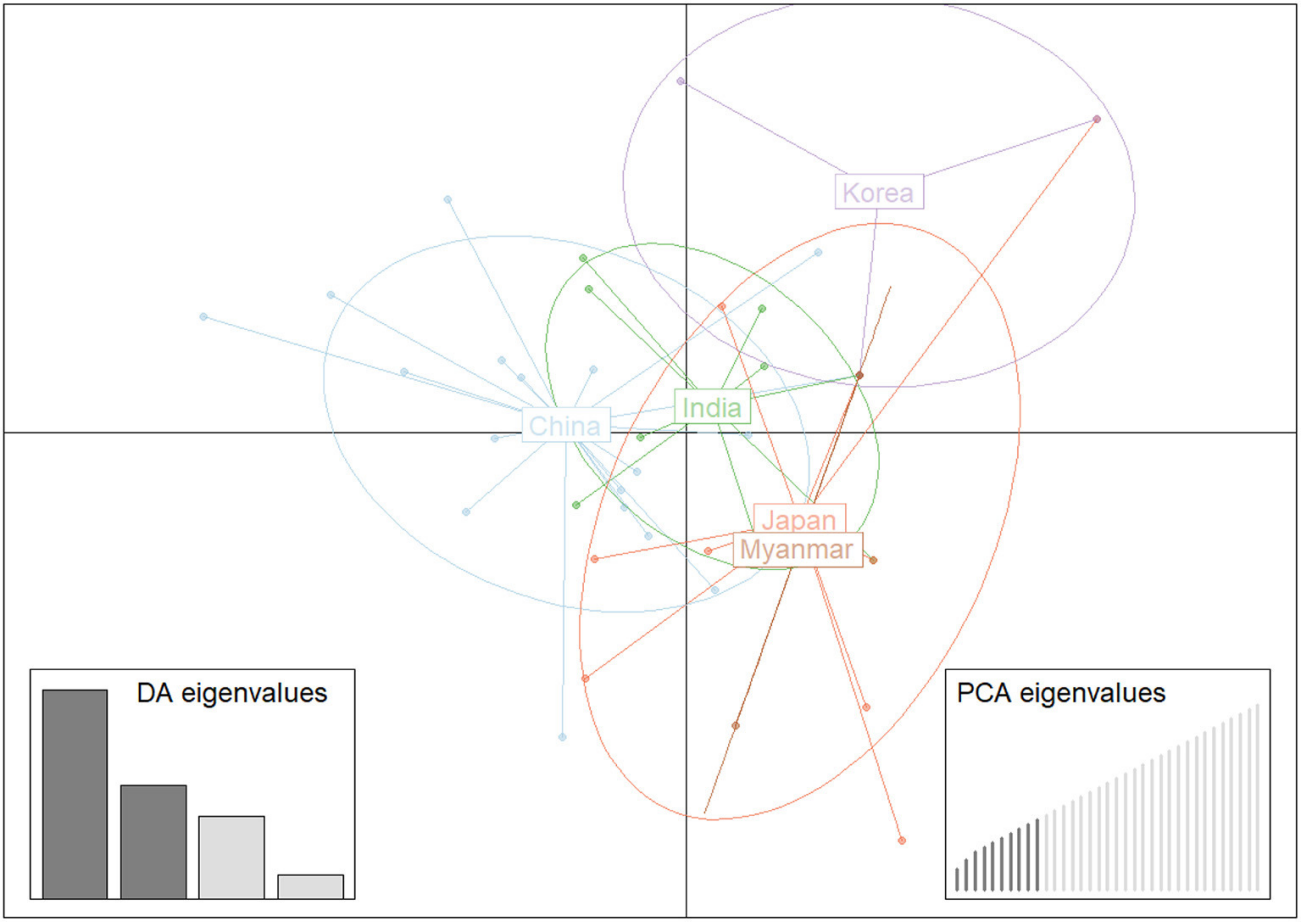
Discriminant analysis of principal components (DAPC) of *Babesia gibsoni* populations in Asia. Each population is pointed out by unique colors.

Structure analysis showed that obvious mixture haplotypes were observed, indicating a weak population structure among populations in Asia, as seen in Figure 2A. Similar results were also found in the genetic structure of *B. gibsoni* populations from Asia, America and Europe (data not shown). Bayesian clustering analysis indicated that there were three distinct clusters (*K* = 3, Figure 2B), suggesting that underlying subgroups were presented in Asia. The DAPC scatterplots analysis showed the geographical distribution characteristics of *B. gibsoni* populations in Asia (Figure 3). The Mantel test indicated no correlation between genetic distance and geographical distance between populations from Asia (*P* = 0.36). These findings suggested a low genetic sub-structuring among *B. gibsoni* populations. Overall, these results were supported by the pairwise *Fst* analysis and AMOVA, with low genetic differentiation among populations.

## Discussion

This study found that the overall nucleotide diversity was 0.0023 and 0.0022 in populations from Asia and the three continents, respectively, revealing the low nucleotide variation among *B. gibsoni* populations. These results were much lower than that of *H*. *canis* (27), but similar to *Plasmodium knowlesi* (55) and *Plasmodium vivax* (56). Haplotype analysis indicated that the genetic diversity was observed with high haplotype diversity in Asian countries except for Bangladesh and India. Low haplotype diversity was found in Bangladesh and India, suggesting that the low degree of genetic variation of these two populations could be related to limited gene communication. The average haplotype diversity of 0.474 and 0.473 were in Asia and the three continents, respectively, which was lower in comparison with other protozoa such as *H*. *canis* (27) and *P. knowlesi* (55). Population size is an essential factor in affecting the genetic diversity, therefore, lower haplotype diversity seemed to correlate with a small effective population size in this study.

Demographic bottleneck is another important factor that can influence the genetic diversity of *B. gibsoni* populations. Neutrality tests showed that the Asian and three continent populations diverged from neutrality, indicating that the two populations probably have experienced recent expansion, which could be caused by bottlenecks or the founder effect (57). Rapid expansion of populations might cause an excess of rare haplotypes as observed in the Asian and three continent populations.

Gene flow is a driving force in the evolution of populations, which can increase the genetic diversity within populations but decrease the genetic difference between populations (58, 59). In this study, the majority of genetic variation in Asia (99.55%) and the three continents (97.90%) were partitioned within populations, implying a high gene flow among these populations as found in other species such as *Cryptosporidium parvum, Giardia duodenalis*, and *H. canis* (27, 60, 61). High gene flow was also supported by the low pairwise *Fst* values, revealing that low genetic differentiation was found between the populations. These results further demonstrated that genetic exchanges occurred among these populations, which could be related to the rapid geographical expansion of ticks and human migration with pets. However, a higher genetic difference was observed between the Chinese population and other populations, which could be correlated with the limited genetic exchanges within Asia. China is one of the largest land area countries with the suitable climate and environment for *H. longicornis* and *R. sanguineus* survival (62, 63), so it can be inferred that the unique ecological system of China prevented the spread of ticks and genetic recombination of *B. gibsoni* with other populations, which would explain why the Chinese population showed a higher genetic differentiation. On a large scale, the highest *Fst* value between the American and European populations indicated low gene flow between populations across continents. Low genetic differentiation was observed between the American and Asian populations, which might be related to the Asian genotype of *B. gibsoni* endemic in America (64). These results were consistent with a previous study in nematode where no or low genetic differentiation was observed between populations from neighboring countries in a single continent but significant genetic differentiation was detected in different continents due to the geographical isolation by distance patterns (65).

Although no correlation between genetic and geographical distances among *B. gibsoni* populations happened (Mantel test, *P* = 0.36), three subgroups were found in Asia that could be supported by Bayesian (*K* = 3) and DAPC analyses. The obvious mixture of haplotypes indicated that the separation among these populations was incomplete and similar findings have been reported in *P. vivax* and *T. annulata* (56, 66). In spite of the existence of ecological and geographical barriers, gene flow between these populations formed a complex population structure, which was probably related to frequent migration of humans carrying asymptomatic animals and the movement of hosts with ticks (27). Ticks and tick-borne diseases could be also carried by migratory birds during breeding and wintering areas as reported previously (67). Therefore, further studies to discover how *B. gibsoni* spread is influenced by human activities or the migration of birds will be essential. Collectively, there are many important factors such as transmission intensity, parasite-host coevolution, geographic and ecological segregation and selective pressure, which can promote the formation of the complex and unique of genetic structure and epidemic patterns among *B. gibsoni* populations.

## Conclusion

In this study, the genetic diversity of *B. gibsoni* was systematically analyzed. These results revealed that low genetic differentiation, high gene flow and high genetic variation within populations were observed among *B. gibsoni* populations from a single continent, but higher genetic differentiation was detected among those across different continents. Neutrality tests indicated that these populations had experienced demographic expansion. These findings will contribute to a comprehensive understanding of genetic diversity of *B. gibsoni* and its complex population structure and provide a reference for developing better control strategies for the public health diseases.

## Data availability statement

The data presented in the study are deposited in the GenBank repository, accession number OM392053-OM392060 and ON810382-ON810384.

## Ethics statement

This study was approved by the Institutional Animal Care and Use Committee of Southwest University. All pet dogs were handled in accordance with the Animal Ethics Procedures and Guidelines of the People's Republic of China. Written informed consent was obtained from the owners for the participation of their animals in this study.

## Author contributions

FY, HY, and FL conceived the project. DL collected blood samples. FY, CG, ZT, DM, and HL carried out laboratory work. FY performed the data analyses and prepared the manuscript with the support from GG and FL. All authors read and approved the final version of the manuscript.

## Supplementary material

Supplementary Figure 1 Haplotype network of *Babesia gibsoni* populations in three continents. Haplotype Hap1 containing 93 *B. gibsoni* isolates from Asia, America, and Europe, respectively. The size of the circle represents the frequency of each haplotype. The different colored dots represent haplotypes from the different populations.

Supplementary Figure 2 Pairwise mismatch distribution analysis of *Babesia gibsoni* populations in Asia. **(A)** Expansion pattern of China population. **(B)** Expansion pattern of Japan population. **(C)** Expansion pattern of Asian population. Red dotted lines represent the observed frequency and blue continuous lines represent the expected frequency.

Supplementary Table 1 Geographical distribution and prevalence of *Babesia gibsoni* in dogs in China.

Supplementary Table 2 The sequences of *Babesia gibsoni* used in this study.
