# Supplementary figures and images for: Analysis of genetic diversity and population structure of Babesia gibsoni
Source: Front Vet Sci. 2023 Mar 23;10:1147958. doi: 10.3389/fvets.2023.1147958 (PMC10076745; doi:10.3389/fvets.2023.1147958)

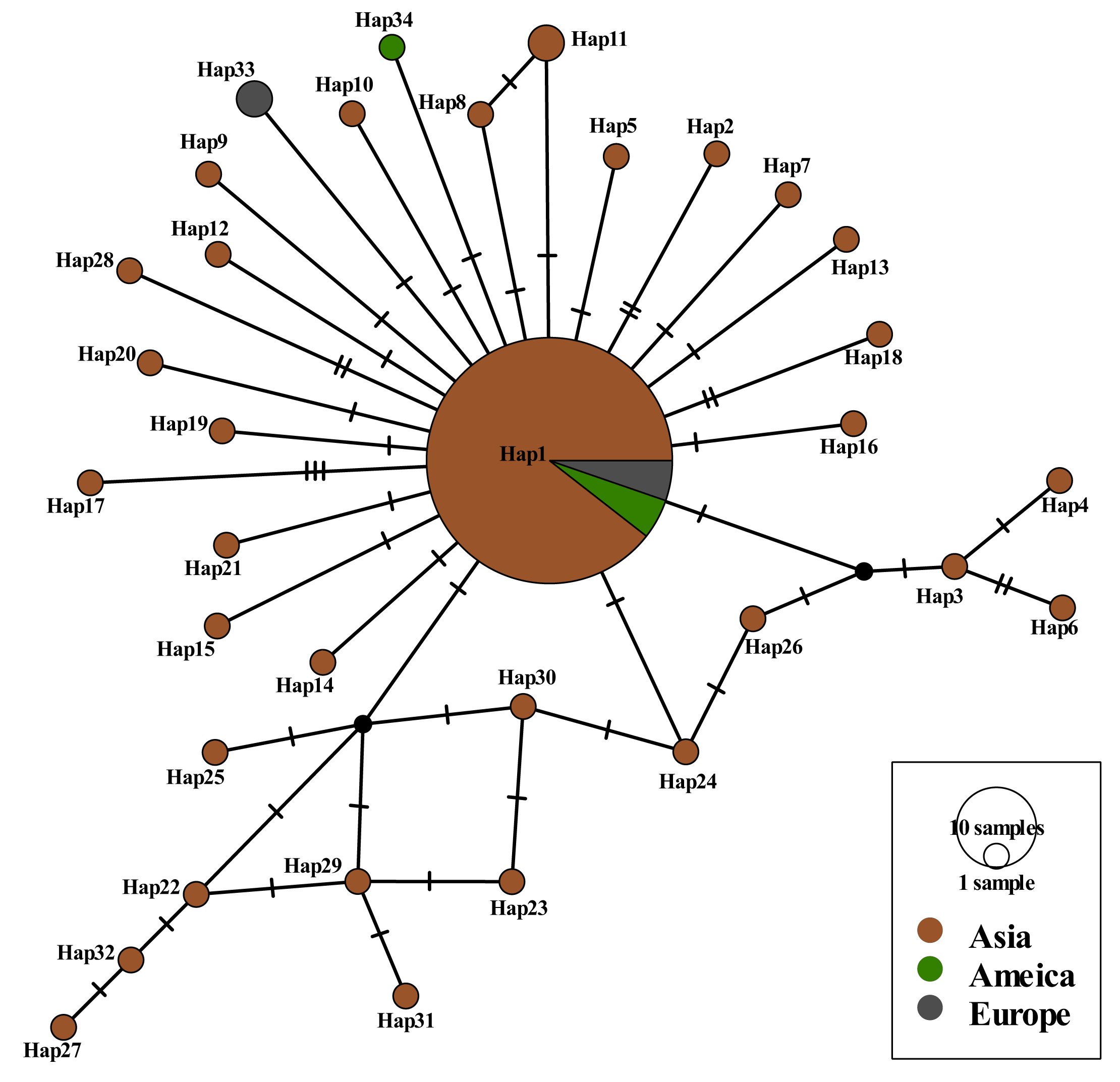

Supplement: Supplementary Figure 1 — Haplotype network of Babesia gibsoni populations in three continents. Haplotype Hap1 containing 93 B. gibsoni isolates from Asia, America, and Europe, respectively. The size of the circle represents the frequency of each haplotype. The different colored dots represent haplotypes from the different populations. [file Image_1.TIF]

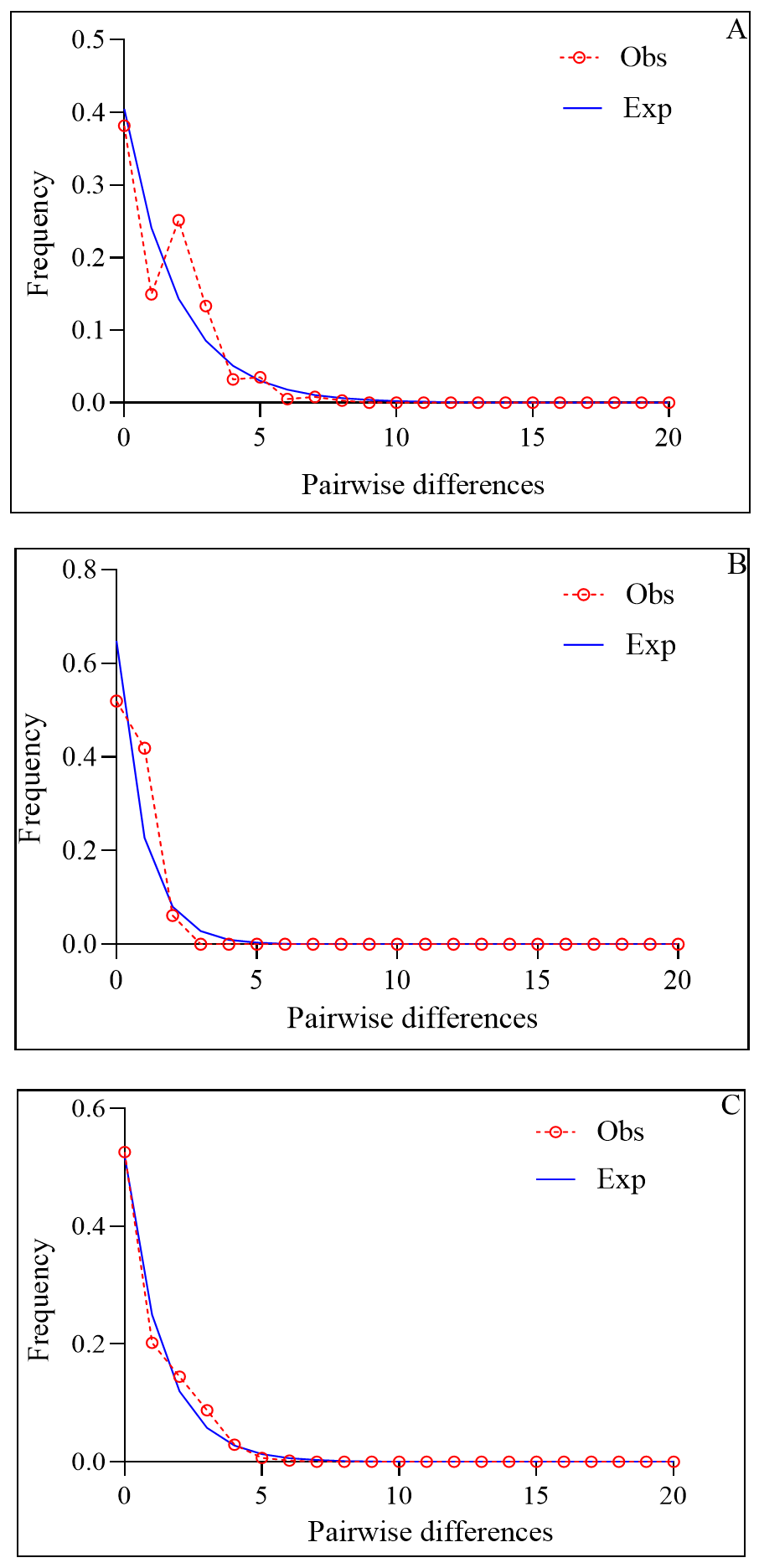

Supplement: Supplementary Figure 2 — Pairwise mismatch distribution analysis of Babesia gibsoni populations in Asia. (A) Expansion pattern of China population. (B) Expansion pattern of Japan population. (C) Expansion pattern of Asian population. Red dotted lines represent the observed frequency and blue continuous lines represent the expected frequency. [file Image_2.TIF]
