# Supplementary material for: Analysis of genetic diversity and population structure of Babesia gibsoni
Source: Front Vet Sci. 2023 Mar 23;10:1147958. doi: 10.3389/fvets.2023.1147958 (PMC10076745; doi:10.3389/fvets.2023.1147958)
Supplement: Supplementary Table 1 — Geographical distribution and prevalence of Babesia gibsoni in dogs in China. [file Table_1.DOCX]

**Table S1** Geographical distribution and prevalence of *Babesia gibsoni* in dogs in China

| Locations | | *N* | *Babesia gibsoni* |
| --- | --- | --- | --- |
| Province/Municipality | City  (longitude, latitude) |  | Positive rate (%, 95%CI) |
| Chongqing | Chongqing  (106.33°E, 29.35°N) | 111 | 3.6 (4/111, 0.99-8.97) |
| Fujian | Quanzhou  (118.37°E, 24.54°N) | 23 | 4.4 (1/23, 0.11-21.95) |
| Hubei | Wuhan  (114.31°E, 30.51°N) | 34 | 14.7 (5/34, 4.95-31.06) |
|  | Xiangyang  (112.14°E, 32.04°N) | 10 | 10.0 (1/10, 0.25-44.50) |
| Shandong | Linyi  (118.32°E, 35.06°N) | 19 | 0 |
| Total |  | 197 | 5.6 (11/197, 2.82-9.77) |
