# Supplementary material for: Analysis of genetic diversity and population structure of Babesia gibsoni
Source: Front Vet Sci. 2023 Mar 23;10:1147958. doi: 10.3389/fvets.2023.1147958 (PMC10076745; doi:10.3389/fvets.2023.1147958)
Supplement: Supplementary Table 2 — The sequences of Babesia gibsoni used in this study. [file Table_2.DOCX]

**Table S2** The sequences of *Babesia gibsoni* used in this study

| Locations | Continents | Accession number | Host | Organ |
| --- | --- | --- | --- | --- |
| Bangladesh | Asia | LC008284 | Dog | Blood |
| Bangladesh | Asia | LC008285 | Dog | Blood |
| Bangladesh | Asia | LC006968 | Dog | Blood |
| China | Asia | MN928814 | Dog | Blood |
| China | Asia | MN928815 | Dog | Blood |
| China | Asia | MN928816 | Dog | Blood |
| China | Asia | MN928817 | Dog | Blood |
| China | Asia | MN928818 | Dog | Blood |
| China | Asia | MN928819 | Dog | Blood |
| China | Asia | MN928820 | Dog | Blood |
| China | Asia | MN928821 | Dog | Blood |
| China | Asia | MN928822 | Dog | Blood |
| China | Asia | MN928823 | Dog | Blood |
| China | Asia | MN928824 | Dog | Blood |
| China | Asia | MN928825 | Dog | Blood |
| China | Asia | MN928826 | Dog | Blood |
| China | Asia | MN928827 | Dog | Blood |
| China | Asia | MN928828 | Dog | Blood |
| China | Asia | MN928829 | Dog | Blood |
| China | Asia | MN928830 | Dog | Blood |
| China | Asia | MN928831 | Dog | Blood |
| China | Asia | MN928832 | Dog | Blood |
| China | Asia | MN928833 | Dog | Blood |
| China | Asia | KP666155 | Dog | Blood |
| China | Asia | KP666156 | Dog | Blood |
| China | Asia | KP666157 | Dog | Blood |
| China | Asia | KP666158 | Dog | Blood |
| China | Asia | KP666159 | Dog | Blood |
| China | Asia | KP666160 | Dog | Blood |
| China | Asia | KP666161 | Dog | Blood |
| China | Asia | KP666162 | Dog | Blood |
| China | Asia | KP666163 | Dog | Blood |
| China | Asia | KP666164 | Dog | Blood |
| China | Asia | KP666165 | Dog | Blood |
| China | Asia | KP666166 | Dog | Blood |
| China | Asia | KP666167 | Dog | Blood |
| China | Asia | KP666168 | Dog | Blood |
| China | Asia | OM392053 | Dog | Blood |
| China | Asia | OM392054 | Dog | Blood |
| China | Asia | OM392055 | Dog | Blood |
| China | Asia | OM392056 | Dog | Blood |
| China | Asia | OM392057 | Dog | Blood |

**Table S2** (continued)

| Locations | Continents | Accession number | Host | Organ |
| --- | --- | --- | --- | --- |
| China | Asia | OM392058 | Dog | Blood |
| China | Asia | OM392059 | Dog | Blood |
| China | Asia | OM392060 | Dog | Blood |
| China | Asia | ON810382 | Dog | Blood |
| China | Asia | ON810383 | Dog | Blood |
| China | Asia | ON810384 | Dog | Blood |
| India | Asia | OK626643 | Dog | Blood |
| India | Asia | OK626644 | Dog | Blood |
| India | Asia | OK626645 | Dog | Blood |
| India | Asia | MN134499 | Dog | Blood |
| India | Asia | MN134500 | Dog | Blood |
| India | Asia | MN134501 | Dog | Blood |
| India | Asia | MN134502 | Dog | Blood |
| India | Asia | MN134503 | Dog | Blood |
| India | Asia | MN134504 | Dog | Blood |
| India | Asia | MN134505 | Dog | Blood |
| India | Asia | MN134506 | Dog | Blood |
| India | Asia | MN134507 | Dog | Blood |
| India | Asia | MN134508 | Dog | Blood |
| India | Asia | MN134509 | Dog | Blood |
| India | Asia | MN134511 | Dog | Blood |
| India | Asia | MN134512 | Dog | Blood |
| India | Asia | MN134513 | Dog | Blood |
| India | Asia | MN134514 | Dog | Blood |
| India | Asia | MN134515 | Dog | Blood |
| India | Asia | MN134516 | Dog | Blood |
| India | Asia | MN134517 | Dog | Blood |
| India | Asia | KJ142323 | Dog | Blood |
| India | Asia | KC461261 | Dog | Blood |
| India | Asia | KF928958 | Dog | Blood |
| India | Asia | KF511955 | Dog | Blood |
| India | Asia | KF511956 | Dog | Blood |
| India | Asia | KF171470 | Dog | Blood |
| India | Asia | KF171471 | Dog | Blood |
| India | Asia | KF171472 | Dog | Blood |
| India | Asia | KF171473 | Dog | Blood |
| India | Asia | KF878944 | Dog | Blood |
| India | Asia | KF878947 | Dog | Blood |
| Japan | Asia | AB118032 | Dog | Blood |
| Japan | Asia | AB478318 | Dog | Blood |
| Japan | Asia | AB478319 | Dog | Blood |
| Japan | Asia | AB478324 | Dog | Blood |

**Table S2** (continued)

| Locations | Continents | Accession number | Host | Organ |
| --- | --- | --- | --- | --- |
| Japan | Asia | AB478325 | Dog | Blood |
| Japan | Asia | AB478326 | Dog | Blood |
| Japan | Asia | AB478327 | Dog | Blood |
| Japan | Asia | AB478328 | Dog | Blood |
| Japan | Asia | AB478329 | Dog | Blood |
| Japan | Asia | AB478330 | Dog | Blood |
| Japan | Asia | AF175300 | Dog | Blood |
| Japan | Asia | LC012792 | Dog | Blood |
| Japan | Asia | LC012793 | Dog | Blood |
| Japan | Asia | LC012794 | Dog | Blood |
| Japan | Asia | LC012795 | Dog | Blood |
| Japan | Asia | LC012796 | Dog | Blood |
| Japan | Asia | LC012797 | Dog | Blood |
| Japan | Asia | LC012798 | Dog | Blood |
| Japan | Asia | LC012799 | Dog | Blood |
| Japan | Asia | LC012800 | Dog | Blood |
| Japan | Asia | LC012801 | Dog | Blood |
| Japan | Asia | LC012802 | Dog | Blood |
| Japan | Asia | LC012803 | Dog | Blood |
| Japan | Asia | LC012804 | Dog | Blood |
| Japan | Asia | LC012805 | Dog | Blood |
| Japan | Asia | LC012806 | Dog | Blood |
| Japan | Asia | LC012807 | Dog | Blood |
| Japan | Asia | LC012808 | Dog | Blood |
| Japan | Asia | LC012809 | Dog | Blood |
| Korea | Asia | AB478320 | Dog | Blood |
| Korea | Asia | AB478321 | Dog | Blood |
| Korea | Asia | AB478322 | Dog | Blood |
| Korea | Asia | AB478323 | Dog | Blood |
| Malaysia | Asia | AF175301 | Dog | Blood |
| Myanmar | Asia | LC602469 | Dog | Blood |
| Myanmar | Asia | LC168620 | Dog | Blood |
| Myanmar | Asia | LC168621 | Dog | Blood |
| St Kitts | America | JX112784 | Dog | Blood |
| USA | America | AF396748 | Dog | Blood |
| USA | America | AF396749 | Dog | Blood |
| USA | America | DQ184507 | Dog | Blood |
| USA | America | EU084677 | Dog | Blood |
| USA | America | EU583386 | Dog | Blood |
| Italy | Europe | MT752609 | Dog | Blood |
| Italy | Europe | MT752610 | Dog | Blood |
| Serbia | Europe | KJ696716 | Dog | Blood |

**Table S2** (continued)

| Locations | Continents | Accession number | Host | Organ |
| --- | --- | --- | --- | --- |
| Serbia | Europe | KJ696717 | Dog | Blood |
| Spain | Europe | AY278443 | Dog | Blood |
